# Survey of Norwegian orthodontists on the use of temporary anchorage devices

**DOI:** 10.1186/s12903-026-08002-5

**Published:** 2026-03-04

**Authors:** Iselin Marie Tostrup, Dimitrios Xenakis, Lars Steinstad, Stein Atle Lie, Maria Mavragani

**Affiliations:** Department of Clinical Dentistry, University of Bergen, Årstadveien 19, Bergen, Bergen, 5009 Norway

## Abstract

**Objectives:**

To assess Norwegian orthodontists’ perspectives concerning use of Temporary Anchorage Devices (TADs).

**Materials and methods:**

A web-based survey (78 questions) was distributed to all 345 members of the Norwegian Association of Orthodontists. Descriptive statistics, cross-tabulations, Chi-square, Fisher’s Exact, or Kruskal-Wallis tests were applied.

**Results:**

Seventy-one orthodontists responded (low response rate of 20.6%). Use of TADs in the buccal/lingual alveolar process, buccal shelf or retromolar pad (ABR) was reported by 41.5%; 29.2% were previous users, 9.2% intended future use, and 20.0% had never used. For palatal TADs, corresponding values were 29.2%, 15.4%, 23.1%, 32.3%; for miniplates: 0%, 10.8%, 21.5% and 67.7%. No associations were found between TAD use and orthodontists’ demographics, clinical experience, or practice type. Common indications included molar mesialization, distalization and posterior intrusion – the latter related to practitioner experience (*P* = 0.041). Frequent complications included screw loosening (91.3%) and soft-tissue overgrowth/irritation (80.4%). Barriers to use included procedural invasiveness and insufficient training, while discontinuation was attributed to complications, chair time, failures, invasiveness and patient discomfort. Over the preceding five years, 23.9% reported increased use, while 43.5% noted a decline. Among current users, satisfaction with ABR and palatal TADs was 69.2% and 70.6%, respectively. The respondents reported reduced treatment time (55.1%) and improved predictability (79%) when TADs were used.

**Conclusions:**

Among responding Norwegian orthodontists, TAD use was reported by a moderate proportion, with a noted decline over the five years preceding the survey. Among users, satisfaction was generally high. All forms of relevant education were identified as key factors in their adoption.

**Supplementary Information:**

The online version contains supplementary material available at 10.1186/s12903-026-08002-5.

## Introduction

Temporary Anchorage Devices (TADs) have precipitated a paradigm shift in orthodontics, gaining widespread clinical adoption in recent years [1]. They are easy to place and demonstrate low failure rates, facilitating their integration into a broad spectrum of clinical applications [2–5]. Nonetheless, complications have been reported, include screw loosening [6], soft tissue irritation [6, 7] and localized infection or peri-implantitis. The extent of clinical acceptance may be influenced by multiple factors, including the practitioners training, perception of invasiveness and complications risk, patient acceptance [8, 9], requirements for anesthesia [10], costs [8], treatment duration [9], availability of specialists for placement, as well as practitioner demographics and clinical experience [11, 12].

Multiple studies have examined orthodontists’ use of TADs [8–23]. A survey of Swedish orthodontists [13] reported that 53.1% used miniscrews. Over half of non-users intended future adoption, space closure was the most common indication, and placements was mostly performed by the orthodontists. Overall, 64.9% reported satisfaction with the success rates. A comparable study of German orthodontists [12] found that 62% used miniscrews and/or palatal implants, although more than 50% used them infrequently (≤ 2 new patients/3 months). Non-users cited lack of suitable clinical indications, skepticism regarding success rates, and the time or complexity of placement as barriers. In Canada [11], 65.8% of orthodontists reported current use of miniscrews, 11.4% had discontinued use, 11.4% planned future use, whereas 11.4% neither used nor intended to use them. Most current users had used them for 6–10 years (30.8%) and over 60% placed the miniscrews themselves. The most common placement-location was the maxillary alveolar buccal process. A study of French orthodontists [15] reported comparable results, with 66.4% currently using miniscrews and 59.6% of non-users considering future adaption. Most users (65%) had treated fewer than 10 patients using miniscrews, and the majority used them infrequently (58.3%). The most common indication was mesialization and/or distalization (87%) and only 32.4% performed the placements themselves. The reported user satisfaction was high (74.5%).

Despite the widespread adoption of TADs in orthodontics, no data on its application among orthodontists in Norway is published. The aim of the present study was to evaluate Norwegian orthodontists’ opinions and experiences concerning TAD use. Along with descriptive data, associations between TAD use and characteristics of the respondents, as well as between complications, failure frequency and several factors recorded were studied.

## Materials and methods

A web-based survey (Appendix 1) assessing the use of TADs among Norwegian orthodontists was distributed by email, via the Norwegian Association of Orthodontists (NKF), to its members. The Regional Committee for Medical and Health Research Ethics (REK) was consulted, but ethical consent was not considered necessary by REK. Nevertheless, the study was registered in the System for Risk and Compliance (RETTE) for projects at University of Bergen.

The survey was developed using Survey-Xact (Rambøll Management Consulting. 2021. Aarhus, Denmark) and comprised 78 items. It was conducted anonymously and designed to take 5–10 min to complete. The variables investigated were respondents’ demographics, TAD-related training and experiences, placement indications and locations, imaging modalities used in treatment planning, type of specialist conducting placement and removal, TAD and force systems used, complications, failures, and user satisfaction. The questionnaire was based on the one developed by Cruz et al. [24], with reference to similar surveys [8–18, 20–23, 25]. Although many questions had been pilot tested in previous studies [11], no formal validation was conducted, nor was a readability evaluation conducted prior to distribution.

The NKF distributed the survey link to its 345 members on April 5th, 2021. A reminder was sent on April 27th, 2021. The data collection period concluded on May 28th, 2021. Data were transferred from the Excel files generated by Survey-Xact to STATA (StataCorp. 2021. Stata Statistical Software: Release 17. College Station, TX: StataCorp LLC) for statistical analysis. Descriptive analysis of the data was performed. Associations between different variables were analyzed using suitable tests. Cross-tabulations were performed for categorical variables, and the significance of observed differences was assessed by the Chi-square tests, or Fisher’s Exact tests (FE) if there were less than 5 expected responses in a cell in the table. Ordinal variables were tested using non-parametric analysis of variance (Kruskal-Wallis). P values of 0.05 or less were considered statistically significant.

## Results

Seventy-one of 345 NKF members responded, for a response rate of 20.6%. Among those, 11 did not answer all questions. The respondents’ characteristics and use of TADs are presented in Table 1. Over the past five years the use of TADs had decreased a lot for 26.1%, while increased a lot for 10.9% (Table 1). No associations were found between changes in TAD usage frequency and the orthodontist’s age, gender, or training in TAD use (all *p* > 0.05; data not shown in table).

**Table 1:** Characteristics of the respondents and their use of TADs

Variable	*n*	%		Variable	*n*	%
Gender				Bracket slot size		
Female	25	35.2		0.018”	14	21.5
Male	45	63.4		0.022”	10	61.5
Other	1	1.4		.018” and 0.022”	11	16.9
**Age (years)**				**Most common TAD indication**		
30–39	13	18.3		Posterior intrusion	1	3.4
40–49	25	35.2		Molar mesialization	22	75.9
50–59	11	15.5		Incisor retraction	2	6.9
> 59	22	31.0		Molar distalization	1	3.4
**Occupation**				Tooth uprighting	1	3.4
Active orthodontist	61	85.9		Impacted tooth traction	2	6.9
Postgraduate student	4	8.5		**Most common TAD complication**		
Retired	6	5.6		Screw loosening	36	78.3
**Practice type (active orthodontists)**				Soft tissue overgrowth/irritation	17	37.0
Solo practice	29	47.5		Infection/peri-implantitis	1	2.2
Group ortho practice	13	21.3		Bleeding	1	2.2
Multi-specialty practice	14	23.0		Osseointegration	1	2.2
Publicly employed	3	4.9		Interference with tooth movement	1	2.2
University	8	13.1		Tooth hypersensitivity	1	2.2
Public dental service (competence centre)	3	4.9		**Observed complications with TADs**		
Other	10	16.4		Screw loosening	42	91.3
**Practice setting (active orthodontists)**				Soft tissue overgrowth/irritation	37	80.4
City with more than 50 000 residents	36	59.0		Infection/peri-implantitis	11	23.9
Town with 5 000 to 50 000 residents	22	36.1		Bleeding	6	13.0
Village with 2 000 to 5 000 residents	3	4.9		Root contact/damage	3	6.5
**Country of specialization**				Screw breakage	5	10.9
Norway	57	80.3		Nasal or sinus perforation	1	2.2
Other European countries	14	19.7		Osseointegration	4	8.7
**Years practiced as an orthodontist**				Migration of TAD	10	21.7
< 2 years	1	1.5		Interference with tooth movement	5	10.9
2–5 years	6	9.0		Slippage into periosteum	1	2.2
6–10 years	16	23.9		**Frequency change in the use of TADs**		
11–15 years	12	17.9		Increased a lot	5	10.9
16–20 years	4	6.0		Increased a little	6	13.0
> 20 years	28	41.8		About the same	15	32.6
**Type of appliances**				Decreased a little	8	17.4
Buccal fixed appliances (metal braces)		77.4		Decreased a lot	12	26.1
Buccal fixed appliances (aesthetic braces)		13.0		**TAD placement locations**		
Lingual appliances		5.6		Buccal alveolar process in the maxilla	34	72.3
Functional appliances		15.5		Palatal alveolar process in the maxilla	14	29.8
Clear aligners		8.2		Buccal alveolar process in the mandible	36	76.6
**Patient types**				Lingual alveolar process in the mandible	1	2.1
Adult patients		16.5		Retromolar pad	5	10.6
Orthognathic surgery cases		24		Mandibular buccal shelf	5	10.6
Patients requiring permanent tooth extractions		9		Hard palate	25	53.2
**Indications for TADs**				Infrazygomatic crest	3	6.4
**Posterior intrusion**				**Most common TAD placement location**		
Routinely		6.9		Buccal alveolar process in the maxilla	3	10.0
Occasionally		37.9		Palatal alveolar process in the maxilla	1	3.3
Non-use		55.2		Buccal alveolar process in the mandible	15	50.0
**Anterior intrusion**				Hard palate	11	36.7
Occasionally		48.3		**Force application with TADs (current users)**		
Non-use		51.7		Directly	11	42.3
**Molar mesialization**				Indirectly	4	15.4
Routinely		27.6		Directly/indirectly	11	42.3
Occasionally		65.5		**Force application with TADs (previous users)**		
Non-use		6.9		Directly	8	40.0
**Incisor retraction**				Indirectly	3	15.0
Routinely		3.5		Directly/indirectly	9	45.0
Occasionally		37.9		**TAD system**		
Non-use		58.6		OrthoEasy^®^ (Forestadent)	19	41.3
**Molar distalization**				BENEfit^®^ (psm Medical Solutions)	13	28.3
Routinely		10.3		3 M™ Unitek™ TAD (3 M Oral Care)	12	26.1
Occasionally		58.6		VectorTAS™ (Ormco)	8	17.4
Non-use		31.0		Spider Screw^®^ (Ortho Technology)	5	10.9
**Tooth uprighting**				The Aarhus^®^ System (American Orthodontics)	4	8.7
Routinely		3.5		tomas^®^ anchorage system (Dentaurum)	3	6.5
Occasionally		34.5		Other: Promedia	1	2.2
Non-use		62.1		**TADs shorten treatment time**		
**Occlusal cant correction**				Strongly agree	5	17.2
Routinely		3.5		Somewhat agree	11	37.9
Occasionally		37.9		Neutral	10	34.5
Non-use		58.6		Somewhat disagree	3	10.3
**Impacted tooth traction**				**TADs have made treatment more predictable**		
Routinely		3.5		Strongly agree	9	31.0
Occasionally		37.9		Somewhat agree	14	48.3
Non-use		58.6		Neutral	3	10.3
**Maxillary expansion**				Somewhat disagree	3	10.3
Occasionally		27.6		**TADs have made treatment better**		
Non-use		72.4		Strongly agree	10	34.5
**Orthopaedics**				Somewhat agree	8	27.6
Occasionally		6.9		Neutral	9	31.0
Non-use		93.1		Somewhat disagree	2	6.9
**Correction of Cl. III**						
Occasionally		31.0				
Non-use		69.0				

The most frequently used placement location was the buccal alveolar process in the mandible, followed by the hard palate (Table 1). Compared with those who had treated 50 or less, orthodontists who had treated more than 50 patients with TADs in the ABR tended to place them more frequently in the following locations: the infrazygomatic crest (FE: *p* = 0.012), buccal shelf (FE: *p* = 0.010), retromolar area (FE: *p* = 0.010), the palatal alveolar process in the maxilla (FE: *p* = 0.001) and buccal alveolar process in the maxilla (FE: *p* = 0.042).

With respect to reported complications (Table 1), orthodontists who had treated more than 50 patients with TADs in the ABR observed infection around the TAD more often than those who had treated 50 or fewer (FE: *p* = 0.007). Current or previous users practicing for more than 10 years experienced less bleeding (FE: *p* = 0.044) and interference with tooth movement (FE: *p* = 0.044) than those practicing for less than 10 years (data not shown in table).

Among the 71 respondents, 43.6% were current TAD users, 25.4% former users, 32.4% non-users with no future plans for usage, and 38.0% intended future use. TADs placed in the buccal/lingual alveolar process, buccal shelf or retromolar pad (ABR) were currently used by 41.5% (Table 2), the majority aged 40–49 (40.7%). Most non-users of TADs in the ABR but with future intentions for use were aged 30–39 and 40–49. TADs in the hard palate were currently used by 29.2% (Table 2), with most in the 30–39 and 40–49 age groups. Of those planning future use of TADs in the hard palate, the majority were aged 40–49. None of the respondents currently used miniplates, though 10.8% were previous users (Table 2); most planning future use were aged 30–39 (35.7%) and 40–49 (35.7%).

**Table 2:** Use of TADs

Variable	ABR		Hard palate		Miniplates	
	*n* %		*n* %		*n* %	
**Use of TADs**						
Yes	27	41.5	19	29.2		
No, but plan to in the future	6	9.2	15	23.1	14	21.5
No, but did at one point	19	29.2	10	15.4	7	10.8
No, never have and don’t plan to	13	20.0	21	32.3	44	67.7
**Years of using TADs (current users)**						
< 2 years	3	11.1	6	33.3		
2–5 years	4	14.8	3	16.7		
6–10 years	10	37.0	5	27.8		
11–20 years	10	37.0	4	22.2		
> 20 years						
**Years of using TADs (previous users)**						
< 2 years	9	50.0	6	60.0	4	66.7
2–5 years	8	44.4	4	40.0	1	16.7
6–10 years	1	5.6			1	16.7
11–20 years						
> 20 years						
**Number of patients treated with TADs**						
< 10	16	35.6	17	60.7	6	100.0
10–20	10	22.2	6	21.4		
21–50	8	17.8	4	14.3		
51–100	6	13.3	1	3.6		
< 100	5	11.1				
**Frequency of use of TADs**						
Very often (> 2 new patients/week)	1	3.7				
Often (> 2 new patients/month)	5	18.5	4	22.2		
Now and then (> 2 new patients/quarter of a year)	11	40.7	2	11.1		
Infrequent (≤2 new patients/quarter of a year)	10	37.0	12	66.7		
**Placement of TADs (Current users)**						
Me	27	100.0	17	94.4		
Periodontist						
Oral surgeon			1	5.6		
Another orthodontist						
General dentist						
**Placement of TADs (Previous users)**						
Me	17	89.5	9	90.0	2	33.3
Periodontist						
Oral surgeon	2	10.5	1	10.0	4	66.7
Another orthodontist						
General dentist						
**Removal of TADs (Current users)**						
Me	27	100.0	17	94.4		
Periodontist						
Oral surgeon			1	5.6		
Another orthodontist						
General dentist						
**Removal of TADs (Previous users)**						
Me	18	100.0	9	90.0	2	33.3
Periodontist						
Oral surgeon			1	10.0	4	66.7
Another orthodontist						
General dentist						
**Training in the use of TADs**						
None	5	7.7	8	12.3	31	47.7
Training in residency	34	52.3	25	38.5	3	4.6
Continuing education lectures	45	69.2	44	67.7	27	41.5
Literature (textbooks and articles)	29	44.6	23	35.4	19	29.2
Hands-on course	37	56.9	32	49.2	7	10.8
Other	3	4.6	2	3.1		
**Satisfaction with success rate (current users)**						
Very satisfied	9	34.6	6	35.3		
Somewhat satisfied	9	34.6	6	35.3		
Neutral	3	11.5	3	17.7		
Somewhat dissatisfied	4	15.4	2	11.8		
Very dissatisfied	1	3.9				
**Satisfaction with success rate (previous users)**						
Very satisfied			3	37.5	2	33.3
Somewhat satisfied	5	31.3	2	25.0		
Neutral	2	12.5	3	37.5	3	50.0
Somewhat dissatisfied	7	43.8			1	16.7
Very dissatisfied	2	12.5				
**Radiographs (current users)**						
None	2	8.0	5	29.4		
Bitewings	6	23.1	2	11.8		
Periapical radiograph	21	80.8	8	47.1		
Panoramic radiograph	21	80.8	8	47.1		
Lateral cephalogram	4	15.4	8	47.1		
CBCT	1	3.8	2	11.8		
Other						
**Radiographs (previous users)**						
None					1	16.7
Bitewings	3	16.7			1	16.7
Periapical radiograph	13	72.2	6	60.0	3	50.0
Panoramic radiograph	14	77.8	4	40.0	4	66.7
Lateral cephalogram	3	16.7	4	40.0	1	16.7
CBCT	1	5.6	1	10.0		
Other			1	10.0		
**Reasons for not using TADs (TAD non-users – never used)**						
Lack of training	6	40.0	12	37.5	28	53.8
Preference for less invasive techniques	9	60.0	19	59.4	31	59.6
Need to administer local anaesthetics	3	20.0	6	18.8	4	7.7
Longer chair time			1	3.1	6	11.5
Many complications			2	6.3	6	11.5
Many failures	4	26.7	2	6.3	2	38.5
Patient refusal/discomfort	3	20.0	7	21.9	9	17.3
Costs	1	6.7	4	12.5	6	11.5
Dissatisfaction with results			1	3.1		
No available specialist					7	13.5
Other	1	6.7	6	18.8	4	7.7
**Reasons for not using TADs (previous users)**						
Lack of training	3	15.8	3	30.0	1	14.3
Preference for less invasive techniques	9	47.4	6	60.0	4	57.1
Need to administer local anaesthetics	3	15.8	2	20.0	1	14.3
Longer chair time	1	5.3	3	30.0	1	14.3
Many complications	8	42.1	2	20.0	2	28.6
Many failures	7	36.8	4	40.0	1	14.3
Patient refusal/discomfort	4	21.1	2	20.0	3	42.9
Costs	1	5.3	1	10.0	2	28.6
Dissatisfaction with results	5	26.3	1	10.0	1	14.3
No available specialist						
Other	2	10.5	2	20.0	1	14.3
**Other anchorage strategies (TAD non-users)**						
Extraoral anchorage	18	48.6	22	47.8	30	47.6
Archwire bends	23	62.2	27	58.7	38	82.6
Fixed auxiliaries	14	37.8	19	41.3	28	44.4
Osseointegrated implants			1	2.2	9	14.3
Inter- and intramaxillary elastics	34	91.9	39	84.8	55	87.3
Other	4	10.8	6	13.0	13	20.6

All current users of TADs in the ABR placed them independently (Table 2). Among former TAD ABR users, 10.5% referred to oral surgeons, citing procedure invasiveness, lack of training, and local anesthesia requirement. Among current users of TADs in the hard palate, 5.6% referred to oral surgeons for placement (Table 2), citing lack of training, local anesthesia requirement, and complication risks. Among former users of TADs in the hard palate, 10.0% referred to oral surgeons, noting procedure invasiveness as reason. Of former users of miniplates, 66.7% referred to oral surgeons (Table 2), citing prolonged chair time, the need for local anesthetics, procedure invasiveness, lack of necessary equipment, and risk for complications as reasons.

Common two-dimensional X-ray techniques were mostly used by the respondents (Table 2). The failure frequency for TADs placed in the ABR was lower for those using periapical radiographs for placement, compared to those who did not (Kruskal-Wallis: *p* = 0.008) (data not shown in table).

The failure frequency for TADs placed in the hard palate was higher for current TAD users who did not use radiographs for placement, compared to those who did (Kruskal-Wallis: *p* = 0.005) (data not shown in table).

Regarding training, 52.3% of respondents using TADs in the ABR had received training during residency. The corresponding values were 38.5% for the hard palate and 4.6% for miniplates (Table 2). Satisfaction with success rate for current and previous users is given in Table 2. Current users were more satisfied with the results of TADs placed in the ABR than previous users (FE: *p* = 0.024, data not shown in table).

Occupational status was associated with the use of TADs in the ABR and miniplates, but only one of the retired orthodontists had used TADs in the ABR (Table 3). Excluding retired orthodontists from the analysis, the association was significant only for miniplates (*p* = 0.020). All postgraduates reported current use of TADs in the ABR, with no prior use of miniplates, although all aimed for future use. Only 19.3% of active orthodontists aimed to use miniplates in the future.

**Table 3:** Associations between different variables and TAD use

Variable	TAD use ABR *p*	TAD use hard palate *p*	Miniplate use *p*
Gender	0.600^A^	0.574 ^A^	0.559 ^A^
Age (years)	0.129 ^A^	0.107 ^A^	0.140 ^A^
Occupation	0.017 ^A^	0.056^A^	0.033 ^A^
Practice type (active orthodontists)			
Solo practice	0.312 ^A^	0.783 ^A^	0.636 ^A^
Group ortho practice	0.747 ^A^	0.311 ^A^	0.692 ^A^
Multi-specialty practice	0.124 ^A^	0.499 ^A^	0.794 ^A^
Publicly employed	1.000 ^A^	0.550 ^A^	1.000 ^A^
University	0.260 ^A^	0.218 ^A^	1.000 ^A^
Public dental service (competence centre)	0.565 ^A^	0.202 ^A^	0.242 ^A^
Other	0.729 ^A^	0.710 ^A^	0.763 ^A^
Practice setting (active orthodontists)	0.587 ^A^	0.140 ^A^	1.000 ^A^
Country of specialization	0.331 ^A^	0.735 ^A^	0.776 ^A^
Years practiced as an orthodontist	0.300 ^B^	0.166 ^B^	0.201 ^A^
Type of appliances			
Buccal fixed appliances (metal braces)	0.097^B^	0.009 ^B^	0.005 ^B^
Buccal fixed appliances (aesthetic braces)	0.067 ^B^	0.213 ^B^	0.206 ^B^
Lingual appliances	0.023 ^B^	0.052 ^B^	0.028 ^B^
Functional appliances	0.462 ^B^	0.029 ^B^	0.067 ^B^
Clear aligners	0.148 ^B^	0.251 ^B^	0.662 ^B^
Patient types			
Adult patients	0.074 ^B^	0.104 ^B^	0.115 ^B^
Orthognathic surgery cases	0.841 ^B^	0.402 ^B^	0.517 ^B^
Patients requiring permanent tooth extractions	0.438 ^B^	0.077 ^B^	0.959 ^B^
Bracket slot size	0.226 ^A^	0.019 ^A^	0.348 ^A^
Training			
None	0.071 ^A^	0.420 ^A^	
Training in residency	0.208 ^A^	0.166 ^A^	
Continuing education lectures	0.102 ^A^	0.085 ^A^	
Literature (textbooks and journal articles)	0.005 ^A^	0.004 ^A^	
Hands-on course	0.001 ^A^	0.015 ^A^	
Indications for TAD use			
Posterior intrusion	0.390 ^**A**^	0.712 ^A^	
Anterior intrusion	1.000 ^**A**^	0.413 ^A^	
Molar mesialization	0.626 ^**A**^	0.062 ^A^	
Incisor retraction	0.149 ^**A**^	1.000 ^A^	
Molar distalization	1.000 ^**A**^	0.174 ^A^	
Tooth uprighting	0.581 ^**A**^	0.161 ^A^	
Occlusal cant correction	0.335 ^**A**^	0.549 ^A^	
Impacted tooth traction	1.000 ^**A**^	0.328 ^A^	
Maxillary expansion	1.000 ^**A**^	0.009 ^A^	
Orthopaedics	1.000 ^**A**^	0.498 ^A^	
Correction of Cl. III	1.000 ^**A**^	0.043 ^A^	
Observed complications			
Screw loosening	1.000 ^A^	0.306 ^A^	
Soft tissue overgrowth/irritation	1.000 ^A^	0.633 ^A^	
Infection/peri-implantitis	0.488 ^A^	1.000 ^A^	
Bleeding	1.000 ^A^	1.000 ^A^	
Root contact/damage	1.000 ^A^	0.345 ^A^	
Screw breakage	0.387 ^A^	0.532 ^A^	
Nasal or sinus perforation	1.000 ^A^	1.000 ^A^	
Osseointegration	1.000 ^A^	0.532 ^A^	
Migration of TAD	0.160 ^A^	0.268 ^A^	
Interference with tooth movement	1.000 ^A^	0.306 ^A^	
Slippage into periosteum	1.000 ^A^	0.385 ^A^	

^A^ Fisher’s Exact test

^B^ Kruskal-Wallis equality-of-populations rank test

Orthodontists who used both .018” and 0.022” slots demonstrated significantly greater use of TADs in the hard palate, while users of solely 0.018” slots tended to not use TADs in that region (Table 3). An association was found between the use of buccal fixed appliances with metal braces and use of TADs (Table 3); non-TAD users without plans for future use used more buccal fixed appliances with metal braces than current users for both TADs in the ABR (Kruskal-Wallis: *p* = 0.020) and the hard palate (Kruskal-Wallis: *p* = 0.005). Additionally, the use of lingual appliances and the use of TADs was found associated (Table 3); respondents using TADs both in the ABR (Kruskal-Wallis: *p* = 0.003) and in the hard palate (Kruskal-Wallis: *p* = 0.012) tended to use more lingual appliances compared to TAD non-users who did not plan to use it. Moreover, an association was found between use of TADs in the hard palate and the use of functional appliances (Table 3), where current users of TADs in the hard palate tended to use more functional appliances than non-users who did not plan to use TADs (Kruskal-Wallis: *p* = 0.021) (data for subgroup analyses not shown in table).

No association was found between TAD use and the percentage of adult patients (Table 3). However, a subgroup analysis revealed that current TAD users treated more adult patients than non-users without intentions for usage (Kruskal-Wallis: ABR: *p* = 0.031, palatal: *p* = 0.023). Years of practice as an orthodontist were not associated with the number of patients treated using TADs (FE *p* > 0.05), nor were years of TAD use related to the orthodontists’ age or gender (FE *p* > 0.05). However, a higher percentage of males (39.3%) had treated more than 50 patients using TADs, compared to females (0%) (FE: *p* = 0.003) (data not shown in table).

Respondents who had received hands-on training or read literature in TAD use reported higher TAD usage (Table 3). Younger orthodontists were more likely to have received training in the use of TADs in the ABR during residency (FE *p* < 0.001) (data not shown in table). In contrast, orthodontists older than 49 were more likely not to have received training in TAD use in both the hard palate (FE: *p* = 0.021) and the ABR (FE: *p* = 0.018) (data not shown in table).

Regarding TAD use and indications (Table 3), the use of TADs in the hard palate was associated with the indication maxillary expansion and correction of Class III malocclusion. Among orthodontists with 10 or fever years of practice, 69.2% did not use TADs for posterior intrusion, whereas 61.5% of those with more than 10 years of experience occasionally did (FE: *p* = 0.041) (data not shown in table).

No associations were found between observed complications and TAD use (Table 3). Among respondents who reported screw loosening, 45.7% described it as common (in 1–10% of cases), and 10.9% as very common (in more than 10% of cases). Soft tissue overgrowth/irritation was reported as common by 41.3% and very common by 6.5%. Other complications were reported as uncommonly (in 0.1-1% of cases), rarely (in less than 0.1% of cases), or never occurring (data not shown in table). A subgroup analysis indicated that all previous users of TADs in the hard palate reported no migration of the TAD, 47.5% of current users reported rare or uncommon occurrence, and 52.9% reported none (FE: *p* = 0.037) (data not shown in table).

Orthodontists who had read literature on TAD use observed TAD migration rarely or uncommonly, while those who had not tended to never observe migration (ABR: FE: *p* = 0.043, palatal: FE *p* = 0.046). Respondents without residency training in the use of TADs in the hard palate rarely or never observed bleeding, while those with such training observed it uncommonly (FE: *p* = 0.047). Orthodontists who had participated in continuous education lectures in the use of miniplates tended to observe osseointegration rarely or uncommonly, while those without such education never observed it (FE: *p* = 0.028) (data not shown in table).

The median percentage of failure frequency of TADs placed in the ABR was higher for previous TAD users compared to current users (Table 4). In the hard palate, failure frequency was higher among those reporting bleeding than those who did not (Kruskal-Wallis: *p* = 0.030), but not for any of the other complications (Kruskal-Wallis: *p* > 0.05) (data not shown in table). No associations were found between failure frequency and training in the use of TADs (Kruskal-Wallis: *p* > 0.05) (data not shown in table).

**Table 4:** Failure frequency and its associations with current and previous users

		ABR		Hard palate		Miniplates	
Variable	**Variable**	% p		% p		% p	
Failure frequency (current users)	Median	15		10			
	Range	0–50		0–50			
Failure frequency (previous users)	Median	30	0.047 ^A^	0	0.072 ^A^	10	
	Range	0–90		0–10		0–40	

^A^ Fisher’s Exact test

## Discussion

This study is the first to assess Norwegian orthodontists’ perspectives on TADs, approximately two decades after their clinical adaptation. Several limitations must be considered, most notably the low response rate (20.6%). The results were based on 71 respondents, of whom approximately 31 reported current or previous TAD use. Surveys of health care professionals report an average response rate of approximately 53% [26]. Comparable orthodontic surveys have reported similar [11] or lower response rates [9, 22, 27], whereas others have reported higher rates [8, 13, 15, 25]. Although web-based surveys are more cost-effective than postal surveys, they typically yield lower response rates [28]. To enhance participation, e-mail reminders were employed, as these are associated with increased response rates [29].

The low response rate may limit representativeness and introduce response bias, potentially affecting the validity of the findings. Orthodontists with greater interest in or experience with TADs may have been more likely to participate, potentially leading to an overestimation of reported TAD use. Consequently, the results should be interpreted as reflecting the practices and attitudes of responding orthodontists rather than the entire population of Norwegian orthodontists. Nevertheless, the study provides valuable national-level insight into TAD use patterns, indications, and perceived challenges—a topic for which empirical data remain limited. Future studies employing alternative recruitment strategies or mixed-methods designs [30] may help increase participation and further substantiate these findings.

Data were collected approximately five years prior to publication. Given the evolving role of skeletal anchorage in orthodontics, current practice patterns may differ from that reflected in this survey, potentially limiting applicability to contemporary practice. It could be estimated that more recently graduated orthodontists use TADs more frequently, and that the recorded intention among ABR users to adopt palatal implants suggests a potential increase in palatal implants use.

The estimated completion time of 5–10 min may have been underestimated for current TAD users, who were required to answer all questions. This could partially explain some incomplete responses, as longer surveys are associated with lower response rates [31]. Mandatory restarting after partial completion and the placement of TAD-specific questions later in the survey may have further contributed to attrition. Questions addressing past TAD use and temporal changes may also have introduced recall bias.

The higher proportion of male respondents may reflect the gender distribution in NKF, although this cannot be verified due to unavailable gender data. In contrast, the majority of NDA members are female [32], suggesting that if NKF follows a similar demographic pattern, male orthodontists may have been overrepresented.

TAD usage was found similar to findings by Meeran et al. [8], but lower than in several other studies [9, 12, 15, 22, 25, 27, 33]. Keim et al. [22] reported an increase in TAD use from 2008 to 2014, followed by a decline in 2020. Notably, in the present study, the use of TADs in the hard palate was low compared with their use in the ABR. The frequency of TAD use in the ABR was generally consistent with findings in France [15] and the “all-around” user values (frequency of miniscrew usage for orthodontists using both miniscrews and palatal implants) reported of German orthodontists [12].

A marked decline in TAD usage over the past five years was observed, surpassing reductions reported in previous studies [11, 22]. This trend may reflect increased clinical experience regarding TADs, facilitating a more selective case selection. Respondents reported a longer duration of TAD use compared to previous studies. The duration of TAD use in the ABR was higher than reported in several prior surveys [9, 13, 15, 23], and slightly higher than reported by Canadian orthodontists [11]. This difference is likely attributed to the fact that three of these studies were published more than five years ago [9, 13, 15]. Additionally, the reimbursement for TADs through Norway’s social security system and the extensive clinical experience of Norwegian orthodontists, many of whom have surgical training as general dentists, may contribute to this.

The respondents reported a shorter duration of TAD use in the hard palate than in the ABR. A high percentage also used TADs in the hard palate for less than two years before discontinuing, citing failure and insufficient training as reasons. This finding is noteworthy, as existing literature suggests lower failure rates in palatal sites compared to both maxillary buccal and mandibular sites [5].

Reasons for not using TADs were a preference for less invasive techniques followed by insufficient training. This aligns with findings from Meeran et al. [8] and Fatani et al. [23], who identified inadequate training as key barriers. A Canadian survey [11] reported both a preference for less invasive techniques and lack of training as key reasons, while Hyde et al. [10] highlighted the requirement for local anesthetics as a deterrent. In the present study, discontinuation was attributed primarily to a preference for less invasive methods and a high incidence of complications and failures. Notably, lack of training was a more frequent concern among those who had never used TADs than among former users, emphasizing the role of education in adoption. Furthermore, non-users treated fewer adult patients, used fewer lingual appliances, and favored metal buccal appliances, indicating a preference for traditional orthodontic approaches.

A greater percentage of respondents reported intentions for future TAD use, compared to those who did not. This differs from the German study [12], who reported the opposite, and differs slightly from the Canadian survey [11]. The reported figures are sustainably lower than a 2008 U.S. survey [9], where the higher percentage may reflect temporal differences. The French study [15] found that most non-users of TADs considered using miniscrews. The increased intention among non-users of TADs in the hard palate to incorporate them may indicate that current users of TADs in the ABR plan to expand their usage. Recent reports on complications and failure rates may have discouraged TAD use [34, 35], despite miniscrew failure rates reported as low as 13.5% [35]. Most former TAD users discontinued use within 6 years and reported a higher percentage of complication and failure rates compared to current TAD users. Early complications and failures may have led to abandonment rather than further education or technique refinements.

Most respondents reported receiving training in TAD use in the ABR during residency, while training in hard palate TADs or miniplates was less common. The extent of residency training among Norwegian orthodontists aligns with recent studies [11, 22]. Buschang et al. [9] found that few American orthodontists had received training in miniscrew placement during residency in 2008, whereas Keim et al. [22] later reported a substantial increase in postgraduate courses on skeletal anchorage among American orthodontists over the past 12 years. The present study also found that younger respondents were more likely to have received residency-based training than older respondents, although no significant association was found between TAD use and age. This may suggest that older orthodontists without residency training later adopted TADs through alternative education.

Most current and previous TAD users placed them independently, consistent with findings from Meeran et al. [8] and Fatani et al. [23]. This percentage is higher compared to several other surveys [9, 10, 12, 13, 15, 17, 25], possibly reflecting Norwegian orthodontists’ prior experience in general dentistry. Orthodontists who did not place TADs in the hard palate themselves referred to oral surgeons for placement, citing lack of training, need for local anesthesia, prolonged chair time, lack of equipment, procedure invasiveness and risk of complications – similar to findings by Hyde et al. [10]. While current ABR TAD users placed them exclusively themselves, former users often referred cases to specialists, which may have increased procedural complexity and costs, potentially contributing to the discontinuation of TAD use in the ABR.

The most frequently used placement site was the mandibular, followed by the maxillary buccal alveolar process and the hard palate, aligning with findings from Canada [11]. This is notable, as failure rates generally are reported higher in the mandibular buccal alveolar process than in the maxilla or the hard palate [36]. Additionally, it was shown that more experienced orthodontists (> 50 cases using TADs in the ABR) placed TADs in more demanding locations such as the infrazygomatic crest, buccal shelf and the retromolar area, likely due to their greater clinical expertise.

Routine TAD indications were molar mesialization (most frequent), molar distalization, and posterior intrusion, consistent with findings by Hyde et al. [10]. This technique is commonly used in the orthodontic management of premolar agenesis. The association between TAD use in the hard palate and its application in maxillary expansion or Class III correction suggests the integration of TADs into more advanced treatment protocols. More experienced orthodontists (> 10 years) more frequently utilized TADs for posterior intrusion, indicating greater proficiency in applying them for more complex movements.

Panoramic and periapical radiographs were used for TAD placement. Both modalities are widely available in orthodontic practices, are cost-effective, and are associated with lower radiation exposure than CBCT. Shirck et al. [17] also reported frequent use of both modalities, while panoramic radiographs were commonly employed by American and Indian orthodontists [9, 10]. The limited use of CBCT by Norwegian orthodontists suggests a prudent and ethically conscious approach to radiographic exposure. However, the findings from this study indicate that the use of radiographic imaging contributes to reduced failure rates, underscoring its importance in optimizing TAD placement.

Most respondents applied forces both directly and indirectly, which is consistent with findings from the Canadian survey [11]. In contrast, an American survey focusing solely on either direct or indirect force application found that most respondents used only direct force application [9].

The most commonly reported complications were screw loosening and soft tissue overgrowth/irritation, the latter potentially contributing to screw loosening [37]. Similar results have been reported in previous studies [8, 11]. An American survey also identified irritation caused by auxiliary springs as a common complication [10], where the majority of respondents utilized direct force application, which may explain their higher prevalence. Although the present study did not assess the anatomical sites of complications, soft tissue overgrowth and irritation are more commonly associated with placement in non-keratinized mucosa [38, 39]. Additionally, TADs exhibit higher success rates when placed in the maxilla compared to the mandible [40].

Orthodontists with greater experience with TADs in the ABR (treating > 50 patients) reported infections around TADs more frequent, likely due to both higher case volumes and greater awareness of complications. More experienced orthodontists (> 10 years) reported fewer instances of bleeding and interference with tooth movement, suggesting that procedural technique improves with clinical experience. The absence of reported TAD migration among previous users, in contrast to current users, may reflect recall bias, fewer cases, or delegation of placement to specialists, thereby reducing direct procedure involvement. These findings may underscore the role of education in the recognition of complications, as greater knowledge appears to be associated with improved detection of adverse-effects.

Current TAD users reported a failure rate of 10–15%, with lower rates in the hard palate, yielding an overall success rate of 85–90%. This is consistent with previous literature on the subject [9, 17, 35, 40, 41]. Compared to current TAD users, former users reported higher failure rates in the ABR but lower failure rates in the hard palate, the latter possibly due to limited use prior to discontinuation. Most respondents agreed that TADs reduce treatment time and enhance treatment predictability and quality, consistent with findings from previous studies [9, 11].

## Conclusions

Among the 71 orthodontists who responded out of 345 invited, a moderate proportion reported using TADs, with a slight decrease in use over the past five years. Non-use was primarily due to lack of training and a preference for less invasive techniques, while previous users cited complications and failures as key reasons for discontinuation. Overall, respondents reported satisfactory TAD success rates, and all forms of relevant education were identified as factors supporting introduction and use of TADs. Given the study’s limitations, findings should be interpreted with caution, and generalization to the entire Norwegian orthodontic population should be avoided.

## Supplementary Information

Supplementary Material 1.

Supplementary Material 2.

## Data Availability

The data underlying this article will be shared upon reasonable request to the corresponding author.
