# Supplementary material for: Survey of Norwegian orthodontists on the use of temporary anchorage devices
Source: BMC Oral Health. 2026 Mar 4;26:642. doi: 10.1186/s12903-026-08002-5 (PMC13067581; doi:10.1186/s12903-026-08002-5)
Supplement: Supplementary file 1 — Supplementary Material 1. [file 12903_2026_8002_MOESM1_ESM.docx]

Appendix 1 English questionnaire

Gender

(1) 🔾 Male

(2) 🔾 Female

(3) 🔾 Do not wish to answer

What is your age?

(1) 🔾 <29 years

(2) 🔾 30-39 years

(4) 🔾 40-49 years

(5) 🔾 50-59 years

(6) 🔾 >59 years

What is your occupational status?

(2) 🔾 Active orthodontist

(3) 🔾 Postgraduate student in orthodontics

(1) 🔾 Retired

Display this question:

If What is your occupational status? = *Active orthodontist*

Or What is your occupational status? = *Retired*

How many years have you practiced as an orthodontist?

(5) 🔾 <2 years

(1) 🔾 2–5 years

(2) 🔾 6-10 years

(3) 🔾 11-15 years

(4) 🔾 16-20 years

(6) 🔾 >20 years

Country of specialization

(1) 🔾 Norway

(2) 🔾 Other European countries

(3) 🔾 Other

Display this question:

If What is your occupational status? = *Active orthodontist*

Practice setting

(1) 🔾 City with more than 50 000 residents

(2) 🔾 Town with 5 000 to 50 000 residents

(3) 🔾 Village with 2 000 to 5 000 residents

(4) 🔾 Village with less than 2 000 residents

Display this question:

If What is your occupational status? = *Active orthodontist*

In what clinical setting do you practice orthodontics? Please select all that apply.

(1) ❑ Solo orthodontic practice

(2) ❑ Group orthodontic practice

(3) ❑ Multi-specialty practice

(4) ❑ Publicly employed orthodontist

(5) ❑ University practice

(6) ❑ Public Dental Service Competence Centre

(7) ❑ Other, describe _____

Display this question:

If What is your occupational status? = *Active orthodontist*

Or What is your occupational status? = *Post-graduate student in orthodontics*

Bracket slot size

(1) 🔾 .018’’

(2) 🔾 .022’’

(3) 🔾 .018’’ and .022’’

(4) 🔾 Other, describe _____

Display this question:

If What is your occupational status? = *Active orthodontist*

Or What is your occupational status? = *Post-graduate student in orthodonticsz*

How many percent of your cases do you treat with each technique?

|  | 0 | 10 | 20 | 30 | 40 | 50 | 60 | 70 | 80 | 90 | 100 |
| --- | --- | --- | --- | --- | --- | --- | --- | --- | --- | --- | --- |
| Buccal braces (metal) | (11) 🔾 | (1) 🔾 | (2) 🔾 | (5) 🔾 | (3) 🔾 | (4) 🔾 | (6) 🔾 | (7) 🔾 | (8) 🔾 | (9) 🔾 | (10) 🔾 |
| Buccal braces (esthetic) | (11) 🔾 | (1) 🔾 | (2) 🔾 | (5) 🔾 | (3) 🔾 | (4) 🔾 | (6) 🔾 | (7) 🔾 | (8) 🔾 | (9) 🔾 | (10) 🔾 |
| Lingual braces | (11) 🔾 | (1) 🔾 | (2) 🔾 | (5) 🔾 | (3) 🔾 | (4) 🔾 | (6) 🔾 | (7) 🔾 | (8) 🔾 | (9) 🔾 | (10) 🔾 |
| Aligners | (11) 🔾 | (1) 🔾 | (2) 🔾 | (5) 🔾 | (3) 🔾 | (4) 🔾 | (6) 🔾 | (7) 🔾 | (8) 🔾 | (9) 🔾 | (10) 🔾 |
| Functional appliances | (11) 🔾 | (1) 🔾 | (2) 🔾 | (5) 🔾 | (3) 🔾 | (4) 🔾 | (6) 🔾 | (7) 🔾 | (8) 🔾 | (9) 🔾 | (10) 🔾 |
| TADs (temporary anchorage devices) | (11) 🔾 | (1) 🔾 | (2) 🔾 | (5) 🔾 | (3) 🔾 | (4) 🔾 | (6) 🔾 | (7) 🔾 | (8) 🔾 | (9) 🔾 | (10) 🔾 |

Display this question:

If What is your occupational status? = *Active orthodontist*

Or What is your occupational status? = *Post-graduate student in orthodontics*

How many percent of your patients

|  | 0 | 10 | 20 | 30 | 40 | 50 | 60 | 70 | 80 | 90 | 100 |
| --- | --- | --- | --- | --- | --- | --- | --- | --- | --- | --- | --- |
| Are adults (18 and above)? | (11) 🔾 | (1) 🔾 | (2) 🔾 | (5) 🔾 | (3) 🔾 | (4) 🔾 | (6) 🔾 | (7) 🔾 | (8) 🔾 | (9) 🔾 | (10) 🔾 |
| Require permanent tooth extractions? | (11) 🔾 | (1) 🔾 | (2) 🔾 | (5) 🔾 | (3) 🔾 | (4) 🔾 | (6) 🔾 | (7) 🔾 | (8) 🔾 | (9) 🔾 | (10) 🔾 |
| Require orthodontics in combination with orthognathic surgery? | (11) 🔾 | (1) 🔾 | (2) 🔾 | (5) 🔾 | (3) 🔾 | (4) 🔾 | (6) 🔾 | (7) 🔾 | (8) 🔾 | (9) 🔾 | (10) 🔾 |

Do you currently use TADs in the buccal/lingual alveolar ridge?

(1) 🔾 Yes

(2) 🔾 No, but I plan to in the future

(3) 🔾 No, but I did at one point

(4) 🔾 No, I never have and don’t plan to in the future

Do you currently use TADs in the hard palate?

(1) 🔾 Yes

(2) 🔾 No, but I plan to in the future

(3) 🔾 No, but I did at one point

(4) 🔾 No, I never have and don’t plan to in the future

Do you currently use miniplates?

(1) 🔾 Yes

(2) 🔾 No, but I plan to in the future

(3) 🔾 No, but I did at one point

(4) 🔾 No, I never have and don’t plan to in the future

What training have you received in TAD placement in the buccal/lingual alveolar ridge, buccal shelf or retromolar pad? Please select all that apply.

(1) ❑ None

(4) ❑ Training in residency

(7) ❑ Continuing education lectures

(5) ❑ Literature (textbooks and journal articles)

(2) ❑ Hands-on course

(6) ❑ Other, describe _____

What training have you received in placement of miniplates? Please select all that apply.

(1) ❑ None

(4) ❑ Training in residency

(7) ❑ Continuing education lectures

(5) ❑ Literature (textbooks and journal articles)

(2) ❑ Hands-on course

(6) ❑ Other, describe _____

What training have you received in placement of miniplates? Please select all that apply.

(1) ❑ None

(4) ❑ Training in residency

(7) ❑ Continuing education lectures

(5) ❑ Literature (textbooks and journal articles)

(2) ❑ Hands-on course

(6) ❑ Other, describe _____

Display this question:

If What is your occupational status? = *Active orthodontist*

Or What is your occupational status? = *Post-graduate student in orthodontics*

AND Do you currently use TADs in the buccal/lingual alveolar ridge, buccal shelf or retromolar pad? = *No, but I plan to in the future*

Or Do you currently use TADs in the buccal/lingual alveolar ridge, buccal shelf or retromolar pad? = *No, I never have and don’t plan to in the future*

Why don’t you currently use TADs in the buccal/lingual alveolar ridge, buccal shelf or retromolar pad? Please select all that apply.

(1) ❑ Lack of training

(3) ❑ Preference for less invasive mechanics

(4) ❑ Need to administer local anesthetic

(5) ❑ Longer chairtime

(6) ❑ Too many complications

(7) ❑ Too many failures

(8) ❑ Patient refusal or discomfort

(9) ❑ Cost

(10) ❑ Disappointed by results

(11) ❑ No specialist available

(12) ❑ Other, describe _____

Display this question:

If What is your occupational status? = *Active orthodontist*

Or What is your occupational status? = *Post-graduate student in orthodontics*

AND Do you currently use TADs in hard palate? = *No, but I plan to in the future*

Or Do you currently use TADs in the hard palate? = *No, I never have and don’t plan to in the future*

Why don’t you currently use TADs in the hard palate? Please select all that apply.

(1) ❑ Lack of training

(3) ❑ Preference for less invasive mechanics

(4) ❑ Need to administer local anesthetic

(5) ❑ Longer chairtime

(6) ❑ Too many complications

(7) ❑ Too many failures

(8) ❑ Patient refusal or discomfort

(9) ❑ Cost

(10) ❑ Disappointed by results

(11) ❑ No specialist available

(12) ❑ Other, describe _____

Display this question:

If What is your occupational status? = *Active orthodontist*

Or What is your occupational status? = *Post-graduate student in orthodontics*

AND Do you currently use miniplates? = *No, but I plan to in the future*

Or Do you currently use miniplates? = *No, I never have and don’t plan to in the future*

Why don’t you currently use miniplates? Please select all that apply.

(1) ❑ Lack of training

(3) ❑ Preference for less invasive mechanics

(4) ❑ Need to administer local anesthetic

(5) ❑ Longer chairtime

(6) ❑ Too many complications

(7) ❑ Too many failures

(8) ❑ Patient refusal or discomfort

(9) ❑ Cost

(10) ❑ Disappointed by results

(11) ❑ No specialist available

(12) ❑ Other, describe _____

Display this question:

If What is your occupational status? = *Active orthodontist*

Or What is your occupational status? = *Post-graduate student in orthodontics*

AND Do you currently use TADs in the buccal/lingual alveolar ridge, buccal shelf or retromolar pad? = *No, but I did at one point*

Why did you stop using TADs in the buccal/lingual alveolar ridge, buccal shelf or retromolar pad? Please select all that apply.

(1) ❑ Lack of training

(3) ❑ Preference for less invasive mechanics

(4) ❑ Need to administer local anesthetic

(5) ❑ Longer chairtime

(6) ❑ Too many complications

(7) ❑ Too many failures

(8) ❑ Patient refusal or discomfort

(9) ❑ Cost

(10) ❑ Disappointed by results

(11) ❑ No specialist available

(12) ❑ Other, describe _____

Display this question:

If What is your occupational status? = *Active orthodontist*

Or What is your occupational status? = *Post-graduate student in orthodontics*

AND Do you currently use TADs in hard palate? *= No, but I did at one point*

Why did you stop using TADs in the hard palate? Please select all that apply.

(1) ❑ Lack of training

(3) ❑ Preference for less invasive mechanics

(4) ❑ Need to administer local anesthetic

(5) ❑ Longer chairtime

(6) ❑ Too many complications

(7) ❑ Too many failures

(8) ❑ Patient refusal or discomfort

(9) ❑ Cost

(10) ❑ Disappointed by results

(11) ❑ No specialist available

(12) ❑ Other, describe _____

Display this question:

If What is your occupational status? = *Active orthodontist*

Or What is your occupational status? *= Post-graduate student in orthodontics*

AND Do you currently use miniplates? = *No, but I did at one point*

Why did you stop using miniplates? Please select all that apply.

(1) ❑ Lack of training

(3) ❑ Preference for less invasive mechanics

(4) ❑ Need to administer local anesthetic

(5) ❑ Longer chairtime

(6) ❑ Too many complications

(7) ❑ Too many failures

(8) ❑ Patient refusal or discomfort

(9) ❑ Cost

(10) ❑ Disappointed by results

(11) ❑ No specialist available

(12) ❑ Other, describe _____

Display this question:

Do you currently use TADs in the buccal/lingual alveolar ridge, buccal shelf or retromolar pad? = *No, but I did at one point*

Or Do you currently use TADs in the buccal/lingual alveolar ridge, buccal shelf or retromolar pad? = *No, I never have and don’t plan to in the future*

Or Do you currently use TADs in the buccal/lingual alveolar ridge, buccal shelf or retromolar pad? = *No, but I plan to in the future*

What other anchorage strategies do you use instead of TADs in the buccal/lingual alveolar ridge, buccal shelf or retromolar pad? Please select all that apply.

(1) ❑ Extra-oral anchorage (i.e. headgear)

(2) ❑ Archwire bends (i.e. tip-back bends)

(3) ❑ Fixed auxiliaries (i.e.lingual arches, pendulum)

(5) ❑ Osseointegrated implants

(7) ❑ Inter- or intramaxillary elastics

(6) ❑ Other, describe _____

Display this question:

Do you currently use TADs in the hard palate? = *No, but I did at one point*

Or Do you currently use TADs in the hard palate? = *No, I never have and don’t plan to in the future*

Or Do you currently use TADs in the hard palate? = *No, but I plan to in the future*

What other anchorage strategies do you use instead of TADs in the hard palate? Please select all that apply.

(1) ❑ Extra-oral anchorage (i.e. headgear)

(2) ❑ Archwire bends (i.e. tip-back bends)

(3) ❑ Fixed auxiliaries (i.e.lingual arches, pendulum)

(5) ❑ Osseointegrated implants

(7) ❑ Inter- or intramaxillary elastics

(6) ❑ Other, describe _____

Display this question:

Do you currently use miniplates? = *No, but I did at one point*

Or Do you currently use miniplates? = *No, I never have and don’t plan to in the future*

Or Do you currently use miniplates? = *No, but I plan to in the future*

What other anchorage strategies do you use instead of miniplates? Please select all that apply.

(1) ❑ Extra-oral anchorage (i.e. headgear)

(2) ❑ Archwire bends (i.e. tip-back bends)

(3) ❑ Fixed auxiliaries (i.e.lingual arches, pendulum)

(5) ❑ Osseointegrated implants

(7) ❑ Inter- or intramaxillary elastics

(6) ❑ Other, describe _____

Display this question:

Do you currently use TADs in the buccal/lingual alveolar ridge, buccal shelf or retromolar pad? = *Yes*

How many years have you been using TADs in the buccal/lingual alveolar ridge, buccal shelf or retromolar pad?

(1) 🔾 <2 years

(2) 🔾 2-5 years

(3) 🔾 6-10 years

(4) 🔾 11-20 years

(5) 🔾 >20 years

Display this question:

Do you currently use TADs in the hard palate? = *Yes*

How many years have you been using TADs in the hard palate?

(1) 🔾 <2 years

(2) 🔾 2-5 years

(3) 🔾 6-10 years

(4) 🔾 11-20 years

(5) 🔾 >20 years

Display this question:

Do you currently use miniplates? = *Yes*

How many years have you been using miniplates?

(1) 🔾 <2 years

(2) 🔾 2-5 years

(3) 🔾 6-10 years

(4) 🔾 11-20 years

(5) 🔾 >20 years

Display this question:

Do you currently use TADs in the buccal/lingual alveolar ridge, buccal shelf or retromolar pad? = *No, but I did at one point*

How many years did you use TADs in the buccal/lingual alveolar ridge, buccal shelf or retromolar pad?

(1) 🔾 <2 years

(2) 🔾 2-5 years

(3) 🔾 6-10 years

(4) 🔾 11-20 years

(5) 🔾 >20 years

Display this question:

If Do you currently use TADs in the hard palate? = *No, but I did at one point*

How many years did you use TADs in the hard palate?

(1) 🔾 <2 years

(2) 🔾 2-5 years

(3) 🔾 6-10 years

(4) 🔾 11-20 years

(5) 🔾 >20 years

Display this question:

If Do you currently use miniplates? = *No, but I did at one point*

How many years did you use miniplates?

(1) 🔾 <2 years

(2) 🔾 2-5 years

(3) 🔾 6-10 years

(4) 🔾 11-20 years

(5) 🔾 >20 years

Display this question:

If Do you currently use TADs in the buccal/lingual alveolar ridge, buccal shelf or retromolar pad? = *Yes*

Or Do you currently use TADs in the buccal/lingual alveolar ridge, buccal shelf or retromolar pad? = *No, but I did at one point*

How many patients have you treated with TADs in the buccal/lingual alveolar ridge, buccal shelf or retromolar pad?

(1) 🔾 <10

(2) 🔾 10-20

(3) 🔾 21-50

(4) 🔾 51-100

(5) 🔾 >100

Display this question:

If Do you currently use TADs in the hard palate? *= Yes*

Or Do you currently use TADs in the hard palate? = *No, but I did at one point*

How many patients have you treated with TADs in the hard palate?

(1) 🔾 <10

(2) 🔾 10-20

(3) 🔾 21-50

(4) 🔾 51-100

(5) 🔾 >100

Display this question:

If Do you currently use miniplates? = *Yes*

Or Do you currently use miniplates? = *No, but I did at one point*

How many patients have you treated with miniplates?

(1) 🔾 <10

(2) 🔾 10-20

(3) 🔾 21-50

(4) 🔾 51-100

(5) 🔾 >100

Display this question:

If Do you currently use TADs in the buccal/lingual alveolar ridge, buccal shelf or retromolar pad? = *Yes*

How often do you insert TADs in the buccal/lingual alveolar ridge, buccal shelf or retromolar pad?

(1) 🔾 Very often (>2 new patients/week)

(2) 🔾 Often (>2 new patients/month)

(3) 🔾 Now and then (>2 new patients/quarter of a year)

(4) 🔾 Infrequent (2 or fewer new patients/quarter of a year)

Display this question:

If Do you currently use TADs in the hard palate? = *Yes*

How often do you insert TADs in the hard palate?

(1) 🔾 Very often (>2 new patients/week)

(2) 🔾 Often (>2 new patients/month)

(3) 🔾 Now and then (>2 new patients/quarter of a year)

(4) 🔾 Infrequent (2 or fewer new patients/quarter of a year)

Display this question:

If Do you currently use miniplates? = *Yes*

How often do you insert miniplates?

(1) 🔾 Very often (>2 new patients/week)

(2) 🔾 Often (>2 new patients/month)

(3) 🔾 Now and then (>2 new patients/quarter of a year)

(4) 🔾 Infrequent (2 or fewer new patients/quarter of a year)

Display this question:

If Do you currently use TADs in the buccal/lingual alveolar ridge, buccal shelf or retromolar pad? = *Yes*

Or Do you currently use TADs in the hard palate? = *Yes*

Or Do you currently use miniplates? = *Yes*

Or Do you currently use TADs in the buccal/lingual alveolar ridge, buccal shelf or retromolar pad? = *No, but I did at one point*

Or Do you currently use TADs in the hard palate? = *No, but I did at one point*

Or Do you currently use miniplates? = *No, but I did at one point*

AND If What is your occupational status? = *Active orthodontist*

Or What is your occupational status? = *Post-graduate student in orthodontics*

How has the frequency of your TAD use changed over the last 5 years?

(1) 🔾 Has increased a lot

(2) 🔾 Has increased a little

(3) 🔾 About the same

(4) 🔾 Has decreased a little

(5) 🔾 Has decreased a lot

Display this question:

If What is your occupational status? = *Active orthodontist*

Or What is your occupational status? = *Post-graduate student in orthodontics*

AND Do you currently use TADs in the buccal/lingual alveolar ridge, buccal shelf or retromolar pad? = *Yes*

Who inserts TADs in the buccal/lingual alveolar ridge, buccal shelf or retromolar pad on your patients? Please select all that apply.

(1) ❑ Me

(2) ❑ Periodontist

(3) ❑ Oral surgeon

(4) ❑ Another orthodontist

(6) ❑ General dentist

(5) ❑ Other, describe _____

Display this question:

If Do you currently use TADs in the buccal/lingual alveolar ridge, buccal shelf or retromolar pad? = *No, but I did at one point*

Who inserted TADs in the buccal/lingual alveolar ridge, buccal shelf or retromolar pad on your patients? Please select all that apply.

(1) ❑ Me

(2) ❑ Periodontist

(3) ❑ Oral surgeon

(4) ❑ Another orthodontist

(6) ❑ General dentist

(5) ❑ Other, describe _____

Display this question:

If What is your occupational status? = *Active orthodontist*

Or What is your occupational status? = *Post-graduate student in orthodontics*

AND Do you currently use TADs in the buccal/lingual alveolar ridge, buccal shelf or retromolar pad? = *Yes*

AND Who inserts TADs in the buccal/lingual alveolar ridge, buccal shelf or retromolar pad on your patients? = *Periodontist*

Or Who inserts TADs in the buccal/lingual alveolar ridge, buccal shelf or retromolar pad on your patients? = *Oral surgeon*

Or Who inserts TADs in the buccal/lingual alveolar ridge, buccal shelf or retromolar pad on your patients? = *Another orthodontist*

Or Who inserts TADs in the buccal/lingual alveolar ridge, buccal shelf or retromolar pad on your patients? = *General dentist*

Or Who inserts TADs in the buccal/lingual alveolar ridge, buccal shelf or retromolar pad on your patients? = *Other*

Why don’t you insert all TADs yourself in the buccal/lingual alveolar ridge, buccal shelf or retromolar pad? Please select all that apply.

(2) ❑ Lack of training

(7) ❑ Need to administer local anesthetic

(1) ❑ Longer chairtime

(5) ❑ Too invasive

(3) ❑ Risk of complications

(4) ❑ Does not have the equipment needed

(6) ❑ Other, describe _____

Display this question:

If Do you currently use TADs in the buccal/lingual alveolar ridge, buccal shelf or retromolar pad? = No, but I did at one point

AND Who inserted TADs in the buccal/lingual alveolar ridge, buccal shelf or retromolar pad on your patients? = *Periodontist*

Or Who inserted TADs in the buccal/lingual alveolar ridge, buccal shelf or retromolar pad on your patients? = *Oral surgeon*

Or Who inserted TADs in the buccal/lingual alveolar ridge, buccal shelf or retromolar pad on your patients? = *Another orthodontist*

Or Who inserted TADs in the buccal/lingual alveolar ridge, buccal shelf or retromolar pad on your patients? = *General dentist*

Or Who inserted TADs in the buccal/lingual alveolar ridge, buccal shelf or retromolar pad on your patients? = *Other*

Why didn’t you insert all TADs yourself in the buccal/lingual alveolar ridge, buccal shelf or retromolar pad? Please select all that apply.

(2) ❑ Lack of training

(7) ❑ Need to administer local anesthetic

(1) ❑ Longer chairtime

(5) ❑ Too invasive

(3) ❑ Risk of complications

(4) ❑ Does not have the equipment needed

(6) ❑ Other, describe _____

Display this question:

If What is your occupational status? = *Active orthodontist*

Or What is your occupational status? *= Post-graduate student in orthodontics*

AND Do you currently use TADs in the hard palate? = *Yes*

Who inserts TADs in the hard palate on your patients? Please select all that apply.

(1) ❑ Me

(2) ❑ Periodontist

(3) ❑ Oral surgeon

(4) ❑ Another orthodontist

(6) ❑ General dentist

(5) ❑ Other, describe _____

Display this question:

If Do you currently use TADs the hard palate? = *No, but I did at one point*

Who inserted TADs in the hard palate on your patients? Please select all that apply.

(1) ❑ Me

(2) ❑ Periodontist

(3) ❑ Oral surgeon

(4) ❑ Another orthodontist

(6) ❑ General dentist

(5) ❑ Other, describe _____

Display this question:

If What is your occupational status? = *Active orthodontist*

Or What is your occupational status? = Post-graduate student in orthodontics

AND Do you currently use TADs in the hard palate? = *Yes*

AND Who inserts TADs in the hard palate on your patients? = *Periodontist*

Or Who inserts TADs in the hard palate on your patients? = *Oral surgeon*

Or Who inserts TADs in the hard palate on your patients? *= Another orthodontist*

Or Who inserts TADs in the hard palate on your patients? *= General dentist*

Or Who inserts TADs in the hard palate on your patients? = *Other*

Why don’t you insert all TADs in the hard palate yourself? Please select all that apply.

(2) ❑ Lack of training

(7) ❑ Need to administer local anesthetic

(1) ❑ Longer chairtime

(5) ❑ Too invasive

(3) ❑ Risk of complications

(4) ❑ Does not have the equipment needed

(6) ❑ Other, describe _____

Display this question:

If Do you currently use TADs in the hard palate? = *No, but I did at one point*

AND Who inserted TADs in the hard palate on your patients? = *Periodontist*

Or Who inserted TADs in the hard palate on your patients? = *Oral surgeon*

Or Who inserted TADs in the hard palate on your patients? = *Another orthodontist*

Or Who inserted TADs in the hard palate on your patients? = *General dentist*

Or Who inserted TADs in the hard palate on your patients? *= Other*

Why didn’t you insert all TADs in the hard palate yourself? Please select all that apply.

(2) ❑ Lack of training

(7) ❑ Need to administer local anesthetic

(1) ❑ Longer chairtime

(5) ❑ Too invasive

(3) ❑ Risk of complications

(4) ❑ Does not have the equipment needed

(6) ❑ Other, describe _____

Display this question:

If What is your occupational status? = *Active orthodontist*

Or What is your occupational status? = *Post-graduate student in orthodontics*

AND Do you currently use miniplates? = *Yes*

Who inserts miniplates on your patients? Please select all that apply.

(1) ❑ Me

(2) ❑ Periodontist

(3) ❑ Oral surgeon

(4) ❑ Another orthodontist

(6) ❑ General dentist

(5) ❑ Other, describe _____

Display this question:

If Do you currently use miniplates? = *No, but I did at one point*

Who inserted miniplates on your patients? Please select all that apply.

(1) ❑ Me

(2) ❑ Periodontist

(3) ❑ Oral surgeon

(4) ❑ Another orthodontist

(6) ❑ General dentist

(5) ❑ Other, describe _____

Display this question:

If What is your occupational status? = *Active orthodontist*

Or What is your occupational status? = *Post-graduate student in orthodontics*

AND Do you currently use miniplates? = *Yes*

AND Who inserts miniplates on your patients? = *Periodontist*

Or Who inserts miniplates on your patients? = *Oral surgeon*

Or Who inserts miniplates on your patients? = *Another orthodontist*

Or Who inserts miniplates on your patients? = *General dentist*

Or Who inserts miniplates on your patients? = *Other*

Why don’t you insert all miniplates yourself? Please select all that apply.

(2) ❑ Lack of training

(7) ❑ Need to administer local anesthetic

(1) ❑ Longer chairtime

(5) ❑ Too invasive

(3) ❑ Risk of complications

(4) ❑ Does not have the equipment needed

(6) ❑ Other, describe _____

Display this question:

If Do you currently use miniplates? = *No, but I did at one point*

AND Who inserts miniplates on your patients? = *Periodontist*

Or Who inserted miniplates on your patients? = *Oral surgeon*

Or Who inserted miniplates on your patients? = *Another orthodontist*

Or Who inserted miniplates on your patients? = *General dentist*

Or Who inserted miniplates on your patients? = *Other*

Why didn’t you insert all miniplates yourself? Please select all that apply.

(2) ❑ Lack of training

(7) ❑ Need to administer local anesthetic

(1) ❑ Longer chairtime

(5) ❑ Too invasive

(3) ❑ Risk of complications

(4) ❑ Does not have the equipment needed

(6) ❑ Other, describe _____

Display this question:

If What is your occupational status? = *Active orthodontist*

Or What is your occupational status? = *Post-graduate student in orthodontics*

AND Do you currently use TADs in the buccal/lingual alveolar ridge, buccal shelf or retromolar pad? = *Yes*

Who removes TADs in the buccal/lingual alveolar ridge, buccal shelf or retromolar pad on your patients? Please select all that apply.

(1) ❑ Me

(2) ❑ Periodontist

(3) ❑ Oral surgeon

(4) ❑ Another orthodontist

(6) ❑ General dentist

(5) ❑ Other, describe _____

Display this question:

If Do you currently use TADs in the buccal/lingual alveolar ridge, buccal shelf or retromolar pad? = *No, but I did at one point*

Who removed TADs in the buccal/lingual alveolar ridge, buccal shelf or retromolar pad on your patients? Please select all that apply.

(1) ❑ Me

(2) ❑ Periodontist

(3) ❑ Oral surgeon

(4) ❑ Another orthodontist

(6) ❑ General dentist

(5) ❑ Other, describe _____

Display this question:

If What is your occupational status? = *Active orthodontist*

Or What is your occupational status? = *Post-graduate student in orthodontics*

AND Do you currently use TADs in the hard palate? = *Yes*

Who removes TADs in the hard palate on your patients? Please select all that apply.

(1) ❑ Me

(2) ❑ Periodontist

(3) ❑ Oral surgeon

(4) ❑ Another orthodontist

(6) ❑ General dentist

(5) ❑ Other, describe _____

Display this question:

If Do you currently use TADs in the hard plate? = *No, but I did at one point*

Who removed TADs in the hard palate on your patients? Please select all that apply.

(1) ❑ Me

(2) ❑ Periodontist

(3) ❑ Oral surgeon

(4) ❑ Another orthodontist

(6) ❑ General dentist

(5) ❑ Other, describe _____

Display this question:

If What is your occupational status? = *Active orthodontist*

Or What is your occupational status? = *Post-graduate student in orthodontics*

AND Do you currently use miniplates? = *Yes*

Who removes miniplates on your patients? Please select all that apply.

(1) ❑ Me

(2) ❑ Periodontist

(3) ❑ Oral surgeon

(4) ❑ Another orthodontist

(6) ❑ General dentist

(5) ❑ Other, describe _____

Display this question:

If Do you currently use miniplates? = *No, but I did at one point*

Who removed miniplates on your patients? Please select all that apply.

(1) ❑ Me

(2) ❑ Periodontist

(3) ❑ Oral surgeon

(4) ❑ Another orthodontist

(6) ❑ General dentist

(5) ❑ Other, describe _____

Display this question:

If Do you currently use TADs in the buccal/lingual alveolar ridge, buccal shelf or retromolar pad? = *Yes*

Or Do you currently use TADs in the hard palate? = *Yes*

Or Do you currently use miniplates? = *Yes*

Or Do you currently use TADs in the buccal/lingual alveolar ridge, buccal shelf or retromolar pad? = *No, but I did at one point*

Or Do you currently use TADs in the hard palate? = *No, but I did at one point*

Or Do you currently use miniplates? = *No, but I did at one point*

Which placement locations do you use when you insert TADs? Please elect all that apply.

(1) ❑ Buccal alveolar process in the maxilla

(2) ❑ Palatal alveolar process in the maxilla

(4) ❑ Buccal alveolar process in the mandible

(3) ❑ Lingual alveolar process in the mandible

(5) ❑ Retromolar pad

(6) ❑ Mandibular buccal shelf

(7) ❑ Hard palate

(8) ❑ Infrazygomatic crest

(9) ❑ Symphyseal area in the mandible

Display this question:

If Do you currently use TADs in the buccal/lingual alveolar ridge, buccal shelf or retromolar pad? = *Yes*

Or Do you currently use TADs in the hard palate? = *Yes*

Or Do you currently use miniplates? = *Yes*

Which placement location do you use most frequently?

(1) 🔾 Buccal alveolar process in the maxilla

(2) 🔾 Palatal alveolar process in the maxilla

(4) 🔾 Buccal alveolar process in the mandible

(3) 🔾 Lingual alveolar process in the mandible

(5) 🔾 Retromolar pad

(6) 🔾 Mandibular buccal shelf

(7) 🔾 Hard palate

(8) 🔾 Infrazygomatic crest

(9) 🔾 Symphyseal area in the mandible

Display this question:

If Do you currently use TADs in the buccal/lingual alveolar ridge, buccal shelf or retromolar pad? = *Yes*

Or Do you currently use TADs in the hard palate? = *Yes*

Or Do you currently use miniplates? = *Yes*

For which applications do you use TADs routinely?

|  | Routinely | Occasionally | Don’t use TADs for this |
| --- | --- | --- | --- |
| Posterior intrusion | (1) 🔾 | (2) 🔾 | (3) 🔾 |
| Anterior intrusion | (1) 🔾 | (2) 🔾 | (3) 🔾 |
| Molar mesialization | (1) 🔾 | (2) 🔾 | (3) 🔾 |
| Incisor retraction | (1) 🔾 | (2) 🔾 | (3) 🔾 |
| Molar distalization | (1) 🔾 | (2) 🔾 | (3) 🔾 |
| Tooth uprighting | (1) 🔾 | (2) 🔾 | (3) 🔾 |
| Occlusal cant correction | (1) 🔾 | (2) 🔾 | (3) 🔾 |
| Impacted tooth traction | (1) 🔾 | (2) 🔾 | (3) 🔾 |
| Maxillary expansion | (1) 🔾 | (2) 🔾 | (3) 🔾 |
| Orthopedics | (1) 🔾 | (2) 🔾 | (3) 🔾 |
| Correction of Cl. III | (1) 🔾 | (2) 🔾 | (3) 🔾 |

Display this question:

If Do you currently use TADs in the buccal/lingual alveolar ridge, buccal shelf or retromolar pad? = *Yes*

Or Do you currently use TADs in the hard palate? = *Yes*

Or Do you currently use miniplates? = *Yes*

Which is your most common indication for the use of TADs?

(1) 🔾 Posterior intrusion

(2) 🔾 Anterior intrusion

(3) 🔾 Molar mesialization

(4) 🔾 Incisor retraction

(5) 🔾 Molar distalization

(6) 🔾 Tooth uprighting

(7) 🔾 Occlusal cant correction

(8) 🔾 Impacted tooth traction

(9) 🔾 Maxillary expansion

(10) 🔾 Orthopedics

(11) 🔾 Correction of Cl. III

Display this question:

If Do you currently use TADs in the buccal/lingual alveolar ridge, buccal shelf or retromolar pad? = *Yes*

What type of imaging do you usually use when planning treatment with TADs in the buccal/lingual alveolar ridge, buccal shelf or retromolar pad? Please select all that apply.

(1) ❑ None

(7) ❑ Bitewing

(3) ❑ Periapical

(2) ❑ Panoramic

(4) ❑ Lateral cephalogram

(5) ❑ CBCT/CT

(6) ❑ Other, describe _____

Display this question:

If Do you currently use TADs in the buccal/lingual alveolar ridge, buccal shelf or retromolar pad? = *No, but I did at one point*

What type of imaging did you usually use when planning treatment with TADs in the buccal/lingual alveolar ridge, buccal shelf or retromolar pad? Please select all that apply.

(1) ❑ None

(7) ❑ Bitewing

(3) ❑ Periapical

(2) ❑ Panoramic

(4) ❑ Lateral cephalogram

(5) ❑ CBCT/CT

(6) ❑ Other, describe _____

Display this question:

If Do you currently use TADs in the hard palate? = *Yes*

What type of imaging do you usually use when planning treatment with TADs in the hard palate? Please select all that apply.

(1) ❑ None

(7) ❑ Bitewing

(3) ❑ Periapical

(2) ❑ Panoramic

(4) ❑ Lateral cephalogram

(5) ❑ CBCT/CT

(6) ❑ Other, describe _____

Display this question:

If Do you currently use TADs in the hard palate? = *No, but I did at one point*

What type of imaging did you usually use when planning treatment with TADs in the hard palate? Please select all that apply.

(1) ❑ None

(7) ❑ Bitewing

(3) ❑ Periapical

(2) ❑ Panoramic

(4) ❑ Lateral cephalogram

(5) ❑ CBCT/CT

(6) ❑ Other, describe _____

Display this question:

If Do you currently use miniplates? = *Yes*

What type of imaging do you usually use when planning treatment with miniplates? Please select all that apply.

(1) ❑ None

(7) ❑ Bitewing

(3) ❑ Periapical

(2) ❑ Panoramic

(4) ❑ Lateral cephalogram

(5) ❑ CBCT/CT

(6) ❑ Other, describe _____

Display this question:

If Do you currently use miniplates? = *No, but I did at one point*

What type of imaging did you usually use when planning treatment with miniplates? Please select all that apply.

(1) ❑ None

(7) ❑ Bitewing

(3) ❑ Periapical

(2) ❑ Panoramic

(4) ❑ Lateral cephalogram

(5) ❑ CBCT/CT

(6) ❑ Other, describe _____

Display this question:

If Do you currently use TADs in the buccal/lingual alveolar ridge, buccal shelf or retromolar pad? = *Yes*

Or Do you currently use TADs in the hard palate? = *Yes*

Or Do you currently use miniplates? = *Yes*

Or Do you currently use TADs in the buccal/lingual alveolar ridge, buccal shelf or retromolar pad? = *No, but I did at one point*

Or Do you currently use TADs in the hard palate? = *No, but I did at one point*

Or Do you currently use miniplates? = *No, but I did at one point*

Which TAD system(s) do you regularly use? Please select all that apply.

(1) ❑ The Aarhus® System (American Orthodontics)

(2) ❑ Dual-Top (RMO)

(3) ❑ Infinitas (Dentsply Sirona)

(4) ❑ OrthoEasy® (Forestadent)

(5) ❑ Spider Screw® (Ortho Technology)

(6) ❑ tomas® anchorage system (Dentaurum)

(7) ❑ 3M™ Unitek™ TAD (3M Oral Care)

(8) ❑ VectorTAS (Ormco)

(10) ❑ BENEfit (psm medical solutions)

(9) ❑ Other, describe _____

Display this question:

If Do you currently use TADs in the buccal/lingual alveolar ridge, buccal shelf or retromolar pad? = *Yes*

Or Do you currently use TADs in the hard palate? = *Yes*

Or Do you currently use miniplates? *= Yes*

Or Do you currently use TADs in the buccal/lingual alveolar ridge, buccal shelf or retromolar pad? *= No, but I did at one point*

Or Do you currently use TADs in the hard palate? = *No, but I did at one point*

Or Do you currently use miniplates? = *No, but I did at one point*

How do you usually apply forces to TADs?

(1) 🔾 Directly

(2) 🔾 Indirectly

(3) 🔾 Both directly and indirectly

Display this question:

If Do you currently use TADs in the buccal/lingual alveolar ridge, buccal shelf or retromolar pad? = *Yes*

Or Do you currently use TADs in the hard palate? = *Yes*

Or Do you currently use miniplates? = *Yes*

Or Do you currently use TADs in the buccal/lingual alveolar ridge, buccal shelf or retromolar pad? = *No, but I did at one point*

Or Do you currently use TADs in the hard palate? = *No, but I did at one point*

Or Do you currently use miniplates? *= No, but I did at one point*

Which of the following complications have you observed in your patients for whom TADs were placed? Please select all that apply.

(1) ❑ Screw loosening

(2) ❑ Soft tissue overgrowth/irritation

(12) ❑ Infection/peri-implantitis

(13) ❑ Bleeding

(11) ❑ Root contact/damage

(9) ❑ Screw breakage

(16) ❑ Nasal or sinus perforation

(15) ❑ Nerve damage

(17) ❑ Osseointegration (difficulty removing TAD)

(5) ❑ Migration of TAD

(6) ❑ Interference with tooth movement

(7) ❑ Tooth hypersensitivity

(10) ❑ Slippage into periosteum

Display this question:

If Do you currently use TADs in the buccal/lingual alveolar ridge, buccal shelf or retromolar pad? *= Ye*s

Or Do you currently use TADs in the hard palate? *= Yes*

Or Do you currently use miniplates? *= Yes*

Or Do you currently use TADs in the buccal/lingual alveolar ridge, buccal shelf or retromolar pad? = *No, but I did at one point*

Or Do you currently use TADs in the hard palate? = *No, but I did at one point*

Or Do you currently use miniplates? = *No, but I did at one point*

For each complication, please specify if you have experienced it never (0% of cases), rarely (<0.1% of cases), uncommonly (0.1-1% of cases), commonly (1-10% of cases), or very commonly (>10% of cases).

|  | Never | Rarely | Uncommonly | Commonly | Very commonly |
| --- | --- | --- | --- | --- | --- |
| Screw loosening | (1) 🔾 | (2) 🔾 | (4) 🔾 | (3) 🔾 | (5) 🔾 |
| Soft tissue overgrowth/irritation | (1) 🔾 | (2) 🔾 | (4) 🔾 | (3) 🔾 | (5) 🔾 |
| Infection/peri-implantitis | (1) 🔾 | (2) 🔾 | (4) 🔾 | (3) 🔾 | (5) 🔾 |
| Bleeding | (1) 🔾 | (2) 🔾 | (4) 🔾 | (3) 🔾 | (5) 🔾 |
| Root contact/damage | (1) 🔾 | (2) 🔾 | (4) 🔾 | (3) 🔾 | (5) 🔾 |
| Screw breakage | (1) 🔾 | (2) 🔾 | (4) 🔾 | (3) 🔾 | (5) 🔾 |
| Nasal or sinus perforation | (1) 🔾 | (2) 🔾 | (4) 🔾 | (3) 🔾 | (5) 🔾 |
| Nerve damage | (1) 🔾 | (2) 🔾 | (4) 🔾 | (3) 🔾 | (5) 🔾 |
| Osseointegration (difficulty removing TAD) | (1) 🔾 | (2) 🔾 | (4) 🔾 | (3) 🔾 | (5) 🔾 |
| Migration of TAD | (1) 🔾 | (2) 🔾 | (4) 🔾 | (3) 🔾 | (5) 🔾 |
| Interference with tooth movement | (1) 🔾 | (2) 🔾 | (4) 🔾 | (3) 🔾 | (5) 🔾 |
| Tooth hypersensitivity | (1) 🔾 | (2) 🔾 | (4) 🔾 | (3) 🔾 | (5) 🔾 |
| Slippage into periosteum | (1) 🔾 | (2) 🔾 | (4) 🔾 | (3) 🔾 | (5) 🔾 |

Display this question:

If Do you currently use TADs in the buccal/lingual alveolar ridge, buccal shelf or retromolar pad? = *Yes*

Or Do you currently use TADs in the hard palate? = *Yes*

Or Do you currently use miniplates? = *Yes*

Or Do you currently use TADs in the buccal/lingual alveolar ridge, buccal shelf or retromolar pad? = *No, but I did at one point*

Or Do you currently use TADs in the hard palate? = *No, but I did at one point*

Or Do you currently use miniplates? = *No, but I did at one point*

Which is your most common complication when using TADs?

(1) ❑ Screw loosening

(2) ❑ Soft tissue overgrowth/irritation

(12) ❑ Infection/peri-implantitis

(13) ❑ Bleeding

(11) ❑ Root contact/damage

(9) ❑ Screw breakage

(16) ❑ Nasal or sinus perforation

(15) ❑ Nerve damage

(17) ❑ Osseointegration (difficulty removing TAD)

(5) ❑ Migration of TAD

(6) ❑ Interference with tooth movement

(7) ❑ Tooth hypersensitivity

(10) ❑ Slippage into periosteum

Display this question:

If Do you currently use TADs in the buccal/lingual alveolar ridge, buccal shelf or retromolar pad? = *Yes*

Or Do you currently use TADs in the buccal/lingual alveolar ridge, buccal shelf or retromolar pad? = *No, but I did at one point*

How many percent of your TADs in the buccal/lingual alveolar ridge, buccal shelf or retromolar pad have failed?

(11) 🔾 0

(1) 🔾 10

(2) 🔾 20

(5) 🔾 30

(3) 🔾 40

(4) 🔾 50

(6) 🔾 60

(7) 🔾 70

(8) 🔾 80

(9) 🔾 90

(10) 🔾 100

Display this question:

If Do you currently use TADs in the hard palate? = *Yes*

Or Do you currently use TADs in the hard palate? = *No, but I did at one point*

How many percent of your TADs in the hard palate have failed?

(11) 🔾 0

(1) 🔾 10

(2) 🔾 20

(5) 🔾 30

(3) 🔾 40

(4) 🔾 50

(6) 🔾 60

(7) 🔾 70

(8) 🔾 80

(9) 🔾 90

(10) 🔾 100

Display this question:

If Do you currently use miniplates? = *Yes*

Or Do you currently use miniplates? = *No, but I did at one point*

How many percent of your miniplates have failed?

(11) 🔾 0

(1) 🔾 10

(2) 🔾 20

(5) 🔾 30

(3) 🔾 40

(4) 🔾 50

(6) 🔾 60

(7) 🔾 70

(8) 🔾 80

(9) 🔾 90

(10) 🔾 100

Display this question:

If Do you currently use TADs in the buccal/lingual alveolar ridge, buccal shelf or retromolar pad? = *Yes*

Or Do you currently use TADs in the buccal/lingual alveolar ridge, buccal shelf or retromolar pad? = *No, but I did at one point*

How satisfied are you with the success rate of your TADs placed in the buccal/lingual alveolar ridge, buccal shelf or retromolar pad?

(1) 🔾 Very satisfied

(2) 🔾 Somewhat satisfied

(3) 🔾 Neutral

(4) 🔾 Somewhat dissatisfied

(5) 🔾 Very dissatisfied

Display this question:

If Do you currently use TADs in the hard palate? = *Yes*

Or Do you currently use TADs in the hard palate? = *No, but I did at one point*

How satisfied are you with the success rate of your TADs placed in the hard palate?

(1) 🔾 Very satisfied

(2) 🔾 Somewhat satisfied

(3) 🔾 Neutral

(4) 🔾 Somewhat dissatisfied

(5) 🔾 Very dissatisfied

Display this question:

If Do you currently use miniplates? = *Yes*

Or Do you currently use miniplates? = *No, but I did at one point*

How satisfied are you with the success rate of your miniplates?

(1) 🔾 Very satisfied

(2) 🔾 Somewhat satisfied

(3) 🔾 Neutral

(4) 🔾 Somewhat dissatisfied

(5) 🔾 Very dissatisfied

Display this question:

If Do you currently use TADs in the buccal/lingual alveolar ridge, buccal shelf or retromolar pad? = *Yes*

Or Do you currently use TADs in the hard palate? = *Yes*

Or Do you currently use miniplates? = *Yes*

Do you find that the use of TADs shortens the treatment time?

|  | Strongly agree | Somewhat agree | Neutral | Somewhat disagree | Strongly disagree |
| --- | --- | --- | --- | --- | --- |
| TADs shorten treatment time | (1) 🔾 | (3) 🔾 | (4) 🔾 | (5) 🔾 | (2) 🔾 |
| TADs have made treatment more predictable | (1) 🔾 | (3) 🔾 | (4) 🔾 | (5) 🔾 | (2) 🔾 |
| TADs have made treatment better | (1) 🔾 | (3) 🔾 | (4) 🔾 | (5) 🔾 | (2) 🔾 |
