# Supplementary material for: Survey of Norwegian orthodontists on the use of temporary anchorage devices
Source: BMC Oral Health. 2026 Mar 4;26:642. doi: 10.1186/s12903-026-08002-5 (PMC13067581; doi:10.1186/s12903-026-08002-5)
Supplement: Supplementary file 2 — Supplementary Material 2. [file 12903_2026_8002_MOESM2_ESM.pdf]

English translation – REK application

Reference number: 2019/208
Project title: Bone Anchorage in Orthodontics
Project leader: Maria Mavragani

Dear Maria Mavragani,

We refer to the submission for assessment received on 21.02.2019. The assessment has been made by the secretariat of REK West.

REK's understanding of the project

The study consists of two parts. In the first part, a review of the literature will be conducted. In the second part, a web-based questionnaire will be sent by email to specialists in orthodontics in Norway.
The questions relate to knowledge and clinical experience regarding the use of bone anchorage devices. The data will be processed anonymously.

Assessment

The Health Research Act applies to medical and health research involving humans, human biological material, or health information, cf. Section 2.
Medical and health research is defined as activity conducted using scientific methodology to obtain new knowledge about health and disease, cf. Section 4.

REK West understands that this project does not aim to obtain new knowledge about health and disease, but rather investigates experiences and practices among orthodontic specialists in Norway.
The project is therefore not subject to application or approval by REK.

Please note that this conclusion is advisory, pursuant to Section 11 of the Public Administration Act.

The committee takes this as information.

Kind regards,
Jessica Svärd
Advisor
Regional Committee for Medical and Health Research Ethics (REK West, Norway)

Original REK application

Vår ref. nr.: 2019/208
Prosjekttittel: Beinforankring i Kjeveortopedi
Prosjektleder: Maria Mavragani

Til Maria Mavragani.

Viser til fremleggingsvurdering innsendt 21.02.2019. Vurderingen er gjort av sekretariatet for REK vest.

REK sin forståelse av prosjektet
Studien består av to deler. I første del skal det gjøres en gjennomgang av litteraturen og i andre del skal en web-basert spørreundersøkelse sendes med e-post til spesialister i kjeveortopedi i Norge. Spørsmålene er relatert til kunnskap og klinisk erfaring ved bruk av beinforankringsapparatur. Data skal behandles anonymt.

Vurdering
Helseforskningsloven gjelder for medisinsk og helsefaglig forskning på mennesker, humant biologisk materiale eller helseopplysninger, jf. hfl § 2. Medisinsk og helsefaglig forskning defineres som virksomhet som utføres med vitenskapelig metodikk for å skaffe til veie ny kunnskap om helse og sykdom, jf. hfl § 4.

REK vest oppfatter at prosjektet ikke søker ny kunnskap om helse- og sykdom, men undersøker erfaringer og praksis blant spesialister i kjeveortopedi i Norge. Prosjektet er dermed ikke søknadspliktig til REK.

Jeg gjør oppmerksom på at konklusjonen er veiledende, jf. forvaltningsloven § 11.

Komiteen tar dette til orientering.

Med vennlig hilsen
Jessica Svärd rådgiver
post@helseforskning.etikkom.no
T: 55978497

Regional komité for medisinsk og helsefaglig forskningsetikk REK vest-Norge (REK vest)
http://helseforskning.etikkom.no

REK
REGIONALE KOMITEER FOR MEDISINSK OG HELSEFAGLIG FORSKNINGSETIKK
